# What is the optimum design for my animal experiment?

**DOI:** 10.1136/bmjos-2020-100126

**Published:** 2021-03-15

**Authors:** Natasha A Karp, Derek Fry

**Affiliations:** 1 Data Sciences and Quantitative Biology, Discovery Sciences, R&D, AstraZeneca R&D Cambridge, Cambridge, UK; 2 The University of Manchester, Manchester, UK

**Keywords:** research design, disease models, animal, models, animal, biostatistics

## Abstract

Within preclinical research, attention has focused on experimental design and how current practices can lead to poor reproducibility. There are numerous decision points when designing experiments. Ethically, when working with animals we need to conduct a harm–benefit analysis to ensure the animal use is justified for the scientific gain. Experiments should be robust, not use more or fewer animals than necessary, and truly add to the knowledge base of science. Using case studies to explore these decision points, we consider how individual experiments can be designed in several different ways. We use the Experimental Design Assistant (EDA) graphical summary of each experiment to visualise the design differences and then consider the strengths and weaknesses of each design. Through this format, we explore key and topical experimental design issues such as pseudo-replication, blocking, covariates, sex bias, inference space, standardisation fallacy and factorial designs. There are numerous articles discussing these critical issues in the literature, but here we bring together these topics and explore them using real-world examples allowing the implications of the choice of design to be considered. Fundamentally, there is no perfect experiment; choices must be made which will have an impact on the conclusions that can be drawn. We need to understand the limitations of an experiment’s design and when we report the experiments, we need to share the caveats that inherently exist.

## Introduction

Concerns over reproducibility, the ability to replicate results of scientific studies, have been raised across virtually all disciplines of research.^1^ Preclinical research has been highlighted as an area that is very susceptible to reproducibility issues.^2^ The poor reproducibility arises not from scientific misconduct^2^ but rather a complex array of interplaying issues. This impacts scientific progress and has significant economic costs.^3^ Unless we address the reproducibility issue, ethical questions arise over the continued use of animals as the harm–benefit balance can be questioned.

The issues impacting reproducibility arise from all stages of the research pipeline including the design, analysis, interpretation and reporting. Within preclinical research, the environment the animals are in, the severity of the procedures they experience, and the interactions they have with animal care staff and experimenters may all affect the quality of the data obtained. The utilisation of in vivo protocol guidelines such as PREPARE^4^ or DEPART^5^ should help reduce the effect of these elements on reproducibility. However, most concerns have been about violation of good research practice^6^ and policy-makers have highlighted that poor training of scientists in experimental design is a significant contributing factor.^2^ At a fundamental level, from a 3Rs (Replacement, Reduction, Refinement) perspective, there is a responsibility to create a robust experimental plan which will ensure that the data collected will answer the biological question. Consequently, we need to embrace the guidelines and checklists and prepare the experiment with the ‘end in sight’. There is no right experimental design for a biological question. Rather there are several choices that must be taken, and these decisions have an impact on the practicality, or the cost, of the experiment and also the conclusions that can be drawn. As there is no perfect experiment for the question being considered, it means we should acknowledge the strengths and weaknesses of our experiments when reporting our results. This has led the Animal Research: Reporting of In Vivo Experiment guidelines (ARRIVE) to require reflection on two aspects of the experiment.^7^ First is the study limitations, with consideration of potential sources of bias and imprecision associated with the result. Second is generalisability, which considers whether the findings of the study are likely to generalise to other species or experimental conditions.

Within this article, we will draw on the current thinking of how to achieve a robust experiment and use two real-world scenarios to explore how the experiments could be run. These two scenarios, involving a laboratory and a non-laboratory in vivo experiment, allows us to explore the most commonly used designs. We have limited the exploration to in vivo research where the model and outcome measure have been selected. This will allow the implications of the choice of designs to be explored as we consider the pros and cons of various approaches. Understanding how different designs vary and how this affects the analysis and subsequent conclusions is critical for recognising the implications of the choices made, and the limitations that arise, and to provide a critical context for the conclusions. We have started the exploration with the classic complete randomised design and then explored the impact of including different design features. We add these features relative to the completely randomised design for simplicity in exploration of the impact. In reality, many experiments can encompass several of these design features simultaneously.

Experiments can become quite complex, particularly with time course studies or hierarchical designs. The statistical analysis implemented is a function both of the hypothesis of interest (the biological question) but also of the experimental design. Consequently, it is important to consider both of these, to ensure that the appropriate statistical analysis is selected for the study. For example, breeding issues often lead to multiple batches of animals within an experiment and in these situations each batch should be considered a block. In this situation, the correct analysis will increase the power. When a design is complex, for example, those that include a time course or hierarchical structure, it becomes harder to ensure the analysis is appropriate. If unsure, we recommend reaching out for statistical support from a professional statistician at the planning stage. This manuscript will help you communicate the planned design to the statistician which will then ensure that the analysis is appropriate and optimal for the biological question of interest.

Throughout the manuscript, we have explored the number of animals to use for the research question of interest. Without loss of generality, and to aid comparison, the exploration of power (sensitivity) is performed assuming the underlying within-animal and between-animal variability is constant across the scenarios. We have therefore selected the sample size (n) by exploring the power as a function of standardised effect (ie, an effect of interest relative to the variability in the data). In addition, we have included guidance on the impact on the power of the different approaches, but accurate comparison is not possible as variability estimates will differ as a function of the design. We have used the Experimental Design Assistant (EDA) as a tool to communicate and visualise the various designs, and to enable us to convey their differences.

## Experimental Design Assistant

**Figure 1:**
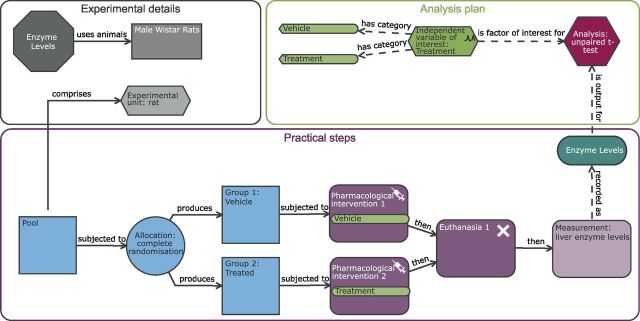
Understanding the EDA diagram. Example of the Experimental Design Assistant (EDA) visualisation. The diagrams are composed of nodes (shapes) and links (arrows). The nodes represent different aspects of an experiment and the links clarify the relationship between different nodes. The diagram consists of three elements: experiment detail (grey nodes), practical steps (blue and purple nodes) and the analysis plan (green and red nodes). Each node can contain additional information which can be accessed within the EDA tool by clicking on the specific node. A two-group completely randomised design is illustrated here.

The EDA is a freely available web-based tool that was developed by the UK National Centre for the Replacement, Refinement and Reduction of Animals in Research (NC3Rs) to guide in vivo researchers through the design and analysis planning of an experiment.^8^ The tool communicates the design via a schematic representation of an experiment and then uses computer-based logical reasoning to provide feedback and advice. A central feature of the EDA was the development of an ontology, a standardised language to communicate experiments (table 1). The variation in language used to define seemingly similar terms can be a significant block to engagement (eg, outcome measure, dependent variable, response variable and variable of interest are all equivalent). For consistency, we have used the same terminology as the EDA throughout this manuscript. Furthermore, the EDA visualisation provides unambiguous representation of different experiments removing any language barrier from understanding the experiment being discussed. Throughout this article, we are using the word treatment to represent the experimental intervention of interest. This ‘treatment’ could be a pharmaceutical treatment, a genetic modification, sex or strain of the animals. Figure 1 shows the standard layout of an EDA diagram. All diagrams created for this manuscript can be imported into the software for future exploration by accessing the.eda files from the Zenodo open access repository.^9^

**Table 1:** Glossary A: central feature of the Experimental Design Assistant (EDA) was the development of an ontology, a standardised language to communicate experiments

Term	Definition
Bias	The overestimation or underestimation of the true effect of an intervention. Bias is caused by inadequacies in the design, conduct or analysis of an experiment, resulting in the introduction of error
Biological unit*	The entity (eg, mouse, cell line) that we would like to draw a conclusion about
Confounder*	A confounder is a nuisance variable that is distributed non-randomly with respect to the independent (treatment) or outcome measure and subsequently can mask an actual association or falsely demonstrate an apparent association
Covariate*	A covariate is a continuous variable that is measurable and considered to have a statistical relationship with the outcome measure
Effect size	Quantitative measure of differences between groups, or strength of relationships between variables
Experimental unit	Biological entity subjected to an intervention independently of all other units, such that it is possible to assign any two experimental units to different treatment groups. Sometimes known as unit of randomisation
External validity	Extent to which the results of a given study enable application or generalisation to other studies, study conditions, animal strains/species or humans
False negative	Statistically non-significant result obtained when the alternative hypothesis is true. In statistics, it is known as the type II error
False positive	Statistically significant result obtained when the null hypothesis is true. In statistics, it is known as the type I error
Independent variable	Variable that the researcher either manipulates (treatment, condition, time), or is a property of the sample (sex) or a technical feature (batch, cage, sample collection) that can potentially affect the outcome measure. Independent variables can be scientifically interesting or can be nuisance variables. Also known as predictor variable
Inference space*	Inference space is the population from which the samples in an experiment were drawn and the population to which results of an experiment can be applied
Internal validity	Extent to which the results of a given study can be attributed to the effects of the experimental intervention, rather than some other, unknown factor(s) (eg, inadequacies in the design, conduct, or analysis of the study introducing bias)
Nuisance variable	Variables that are not of primary interest but should be considered in the experimental design or the analysis because they may affect the outcome measure and add variability. They become confounders if, in addition, they are correlated with an independent variable of interest, as this introduces bias. Nuisance variables should be considered in the design of the experiment (to prevent them from becoming confounders) and in the analysis (to account for the variability and sometimes to reduce bias). For example, nuisance variables can be used as blocking factors or covariates
Observation unit*	The entity on which measurements are made
Outcome measure	Any variable recorded during a study to assess the effects of a treatment or experimental intervention. Also known as dependent variable, response variable
Power	For a predefined, biologically meaningful effect size, the probability that the statistical test will detect the effect if it exists (ie, the null hypothesis is rejected correctly)
Sample size (n)	Number of experimental units per group, also referred to as n
N	Total number of animals used within an experiment

We have therefore used the EDA terminology and definitions^78^ for consistency. Terms with the * were not defined by the EDA-associated literature.

## How experimental designs differ

The goal of an experiment is to explore cause and effect. The resulting experimental process relies on us manipulating a variable (the independent variable of interest) to see what effect it has on a second variable (the outcome measure). We seek to design experiments that have high internal validity, meaning we have confidence that we have eliminated alternative explanations for a finding. Pivotal to achieving high internal validity is the use of randomisation, where test subjects are randomly and independently assigned to ‘treatment’ groups. The need for randomisation should also be considered in the practical steps of the experiments (application of treatment, sample processing and measurement etc) to minimise potential order effects. Randomisation is a necessary process to minimise the risk of nuisance variables impacting the ability to draw conclusions about cause and effect by ensuring that potential nuisance variables are equally distributed across the test groups. Without randomisation, confounding can occur. An experiment is confounded when unintentionally the test groups differ (accidental bias) for a variable that also alters the outcome of interest. When we are trying to isolate a treatment effect, this accidental bias can mask a treatment effect of interest or it can erroneously imply a treatment effect.

The importance of randomisation can be seen in meta-analyses which found that studies that did not randomise were more likely to detect a treatment effect (OR 3.4)^10^ and overinflate the estimated treatment effect.^11 12^ Different designs often differ in how randomisation is implemented.

A component of the randomisation process is the experimental unit (EU), which is the entity which can be assigned independently, at random, to a treatment.^13^ Therefore, any two EUs must be capable of receiving different treatments. Correctly identifying the experimental unit is pivotal to correctly implementing a design, analysing the data and ensuring sufficient data are collected for the biological goals. The challenges of correctly identifying the EU has led to the introduction of three concepts that can characterise an experiment: biological unit (BU), EU and observation unit (OU).^14^ The BU is the entity (eg, mouse, cell line) that we would like to draw a conclusion about. The EU is the entity (eg, animal, cage, litter) that is randomly and independently assigned to the treatment. Often the BU and EU are the same entity. Finally, it is worth considering the OU which is the entity on which measurements are made (eg, tumour (volume), liver (size), blood sample (cell count)). When there are multiple OUs for an EU, there is a risk of analysis errors as the user may fool the analysis into thinking there are more independent observations (or pieces of information) then there actually are. This can lead to false-positive conclusions. Furthermore, the users may incorrectly replicate the wrong element of the experiment if the researcher treats the number of OUs as the N. For each of the designs we describe below, we will consider the three concepts. This will assist us in deducing the inference space of the study correctly, to replicate at the right level of the design and for correctly implementing a design. Further examples to help correctly identify the BU, EU and the OU are explored by Lazic *et al*.^14^

Standardisation is another tool used alongside randomisation to minimise the effect of nuisance variables on studies. Here, a researcher controls potential nuisance variables by standardising all aspects of the research experiment. Within in vivo research, it is common to standardise the animals (eg, inbred animals of one sex), the environment (eg, standard housing and husbandry conditions), the testing procedure and time (eg, conducting the experiment in one batch at the same time). The driver behind standardisation is not just to manage potential confounders but also to reduce the variation in the data, with the argument that this will result in fewer animals being needed to detect a defined effect size of interest. The reality of experimental design is that we have to simplify a complex world into a testing space where we have isolated cause and effect such that there is confidence that the effect seen is arising from the treatment. This process generates an inference or testing space, which relates to the population sampled. Following these experiments, we then draw conclusions and generalise the results to a broader population. External validity is the extent to which the results of a study can be generalised to another population. The complexity in biology means we do have to make hard choices when we design experiments; we cannot explore the impact of all sources of variation on the treatment effect. To progress our scientific understanding, we therefore need to generate ‘do-able problems’^15^ allowing us to unravel the biological story incrementally. Different designs will have different external validity and thus differ in the conclusions that can be drawn.

Different designs also differ in the statistical analysis that is suitable. Statistical analysis is an essential tool to query the data and assess whether the differences seen are likely to arise from sampling effects or an underlying population difference. The majority of animal experiments use hypothesis-testing (with statistical tests such as Student’s *t*-test) and compare the resulting p value against a p*-*value threshold (typically 0.05) to assess whether a statistically significant treatment effect occurred. This process inherently has a risk of false-positive (type I) and false-negative (type II) errors. If the data analysis does not sufficiently account for the study design nor the characteristics of the data (eg, whether it is normally distributed), “the conclusion will be wrong except by accident”.^16^ This is because the assumptions of the statistical test would not be met and the false-positive error rate will no longer be controlled.

Experimental design strategies can be used to manage variation in the data with the goal of increasing the statistical power. The statistical power is the probability that the statistical test will detect the treatment effect if it exists for a predefined effect size of interest. Statistical power is therefore lower when the false-negative error rate is higher. Historically, we have focused on minimising false positives rather than ensuring we had sufficient statistical power. This approach fails to consider that a series of experiments with low power results in false-positive errors dominating.^17 18^ When low-powered experiments are run, we are missing many findings and also the findings being published are more likely to be false. This raises ethical questions about not making proper use of the animals and a wastage which is not in keeping with the Reduction element of the 3Rs. Furthermore, with small effects, statistical significance will only be achieved when sampling differences leads to the effect size being overestimated or the variance underestimated; an effect described as the winner’s curse.^19 20^ For both scientific progress and ethical use of animals, it is important that experiments are adequately powered.

## Case study 1: rat liver study

The key biological question is to explore the effect of compound X on apoptosis in the liver. The compound of interest has been found to produce aggression with rodents, which will be single housed to avoid welfare issues. Practically, after treatment, the rats will be euthanised and the liver harvested. The biological question will be studied by quantifying histological effects in the liver. A cross-section of each liver will be prepared for histological assessment and the number of apoptotic cells counted. Using this case study, we explore a variety of experimental designs and consider their pros and cons.

### Completely randomised designs (CRDs)

In a CRD, the treatments are assigned completely at random so that each experimental unit has the same chance of receiving any one treatment. For this type of design, any difference among experimental units receiving the same treatment is considered as experimental error. This is the simplest design that can be implemented and could be considered the building block for other designs. Critical to this design is the randomisation process. Through the random allocation of experimental units to the treatment groups, the experimenter can assume that, on average, the nuisance variables will affect treatment conditions equally; so, any significant differences between conditions can fairly be attributed to the treatment of interest.

**Figure 2:**
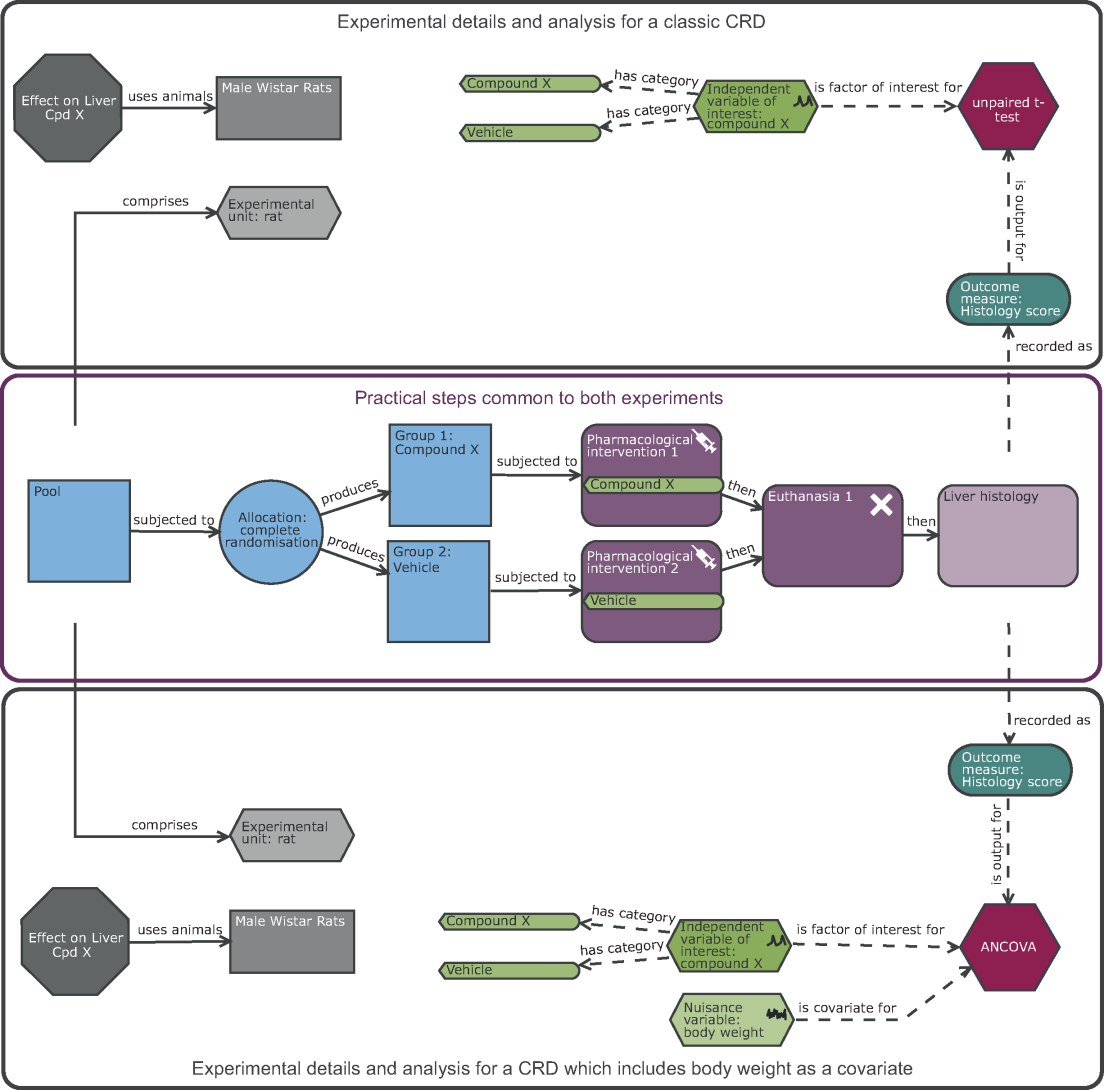
Schematic of a completely randomised design (CRD) of a rat liver study exploring the effect of compound X on the histological score either as a classic experiment or as an experiment which includes a covariate. The purple section highlights the common practical steps: In a complete randomised design experiment, there is one factor of interest, and in this scenario it is treatment which has two possible levels (vehicle or compound X). The animals are the experimental units and form a pool which are randomly allocated to the two treatment groups prior to exposure. In this design, the male Wistar rat is the biological unit, experimental unit and observation unit. The upper black section details the experimental and analysis details for a classic CRD while the lower black section details the CRD with a covariate of pre-treatment body weight and hence includes in the analysis the nuisance variable body weight. The inclusion of the pre-treatment body weight will increase the power if body weight is correlated to the outcome measure. The inclusion subtly impacts the conclusion in that the estimated treatment effect would be the change in means after adjusting for any differences in pre-treatment body weight.

Consider the liver case study: with a completely randomised design (figure 2), we would be using a robust simple design with simple statistical analysis. This can be seen in that the BU is equivalent to the EU and the OU; the male Wistar rat. The experiment was conducted to minimise variation by implementing a standardised protocol with one operator working with one batch of animals. The conclusions drawn will therefore relate to the population sampled, which is male Wistar rats housed in a highly standardised environment. The testing space is therefore narrow and consequently the external validity is low.

A power analysis is a recommended strategy for determining the sample size (n, the number of animals per group) and hence the N (total number of animals) needed for an experiment. Once the design and subsequent analysis plan is established, there are four factors that affect power: the magnitude of the effect (effect size), the variability in the outcome measure, the significance level (typically set at 0.05) and the number of measures per group (n). At a minimum, the statistical power should be at least 0.8. For a confirmatory study (a study performed to confirm an earlier finding), a higher power should be used as the risk of a false negative has more impact on the research than in a hypothesis-generating study. For many designs, formulae exist to complete the power calculation and have been enabled in freeware such as Gpower,^21^ InVivoStat,^22^ or Russ Lenth Power and Sample Size Calculator.^23^ Many researchers struggle to select the effect size of interest to use, in these cases the concept of minimally important difference (MID),^24^ the effect size that would have a biological meaningful impact in the system being studied, is helpful. The researcher needs to determine what size of difference would be an interesting effect that would be worth sharing with the community. If you have multiple variables of interest and cannot prioritise or there are no data to estimate the variability, one option is to consider ‘rules of thumb’ or norms for a study type.^24^ For a two-group study with a continuous outcome measure, Cohen’s *d* is one such approach. It is a standardised effect size, considering the change in mean relative to the SD. Cohen explored overlap of distribution, percentage of variance and correlation to propose 0.2, 0.5 and 0.8 as small, medium and large effect sizes, respectively, in the field of behavioural science.^25^ The case study here is a toxicology study and the markers have been selected as they will have a marked response to damage. Consequently, the European Food Safety Authority have provided guidance on the design of repeat dose toxicity study and highlighted that a difference equivalent to 1 SD was considered of little toxicological relevance.^26^ For the rat case study, we have no pilot data to drive the power calculation, so we will instead power the experiment to detect an effect size expressed as a change relative to the SD.^24^ As this is a toxicology study, we have selected a large effect size of 1.5× the SD as the MID and this effect size would require eight animals per group (Russ Lenth Power Tool,^23^ SD 1, significance threshold=0.05, tails=two, effect size=1.5, power=0.7965).

### Completely randomised designs including covariates

Although strictly speaking not part of the experimental design, a covariate is a continuous variable that is measurable and has a statistically significant relationship with the outcome measure. Examples include body weight, organ weight, tumour size at point of randomisation, pre-treatment activity levels, baseline blood pressure measures and so on. Including a covariate is a noise reduction strategy and consequently has the potential to increase the power of your experiment. When the variable is omitted from the analysis, any variation that arises from this variable is classed as unexplained variation and this therefore decreases the power when the effect of interest is compared with the unexplained variation. To increase the statistical power of an experiment, experimenters typically focus on the n, as for most scenarios there is limited ability to influence the effect size nor the variability in the data. Inclusion of a covariate is an example where alteration of the analysis rather than the design is an alternative path to increasing power. Research has found the inclusion of a covariate is most beneficial when the covariate is strongly correlated with the outcome measure in the population, and when the experimental design would have been only moderately powered (40%–60%) without including the covariate in the analysis.^27^ Including a covariate in the analysis does add additional assumptions that need exploring to ensure the conclusions are robust. The analysis will assume the relationship between the outcome measure and covariate is linear and the relationship is independent of the treatment effect.

Including a covariate might also be necessary to remove a confounder which could not be removed by standardisation or randomisation. The removal is key to achieve internal validity and have greater confidence in the biological conclusion being drawn. A common example in biomedical research arises when the treatment induces a body weight phenotype and the outcome measure correlates with body weight. For example, as body weight is a highly heritable trait, 30% of knockout lines have a body weight phenotype.^28^ As many variables (eg, grip strength, bone density, heart weight) correlate with body weight, it is unsurprising when a gene knockout results in a difference in these associated variables. The question is whether the change in these variables is greater than expected given the observed change in body weight. Many researchers use ratio correction (often called normalisation) to try and adjust for this. Unfortunately, this method is flawed as the underlying assumptions of this process are unlikely to be met and can lead to erroneous conclusions.^29^ The recommendation is to include the nuisance variable that is confounding the study as a covariate in analysis such that you can isolate the treatment effect on the mean readings after adjusting for the confounding relationship. The analysis can be supplemented with additional visualisation to assess the assumptions that arise from including a covariate.^29^

Consider the liver case study: with a completely randomised design, we could include the covariate of pre-treatment body weight (figure 2). As we have no prior knowledge of the correlation strength between the outcome measure and body weight, we could follow the recommendations of Wang *et al*^27^ to conduct analysis both with and without the covariate and infer the presence of an effect if either test is significant. Wang *et al* argued that this approach has a minor inflation impact on the false-positive error rate but it can significantly increase the power. Therefore, we will power the experiment based on the CRD using an n of 8 and enjoy the potential power boost if it arises. This adaption to the design has no impact on the EU or OU. The BU is still the male Wistar rat, but the inclusion of pre-treatment body weight has a small impact on the conclusion as the estimated treatment effect would be the change in means after adjusting for any differences in pre-treatment body weight.

### Factorial designs

In experimental design, a factor is another name for a categorical independent variable. It is a variable that is manipulated by the experimenter and will have two or more levels (eg, light levels could have three levels such as dark, 500 lux or 1000 lux). Examples could be related to animal characteristics (eg, sex, strain, age) or aspects of the environment (eg, environmental enrichment, group size) or aspects of the protocol (eg, timings of measurement, delivery route, dose level). A factorial design investigates the impact of changes in two or more factors within a single experiment. In contrast to one-variable-at-a-time studies, factorial experiments are more efficient (provide more information and/or use fewer experimental units) and allow us to explore the interactions of the factors.^30^ Interaction effects occur when the effect of one variable depends on the value of another variable. For example, in a study of the effect of a drug on blood pressure measurements using the three treatment levels of vehicle, low dose and high dose, we can simultaneously explore the effect of delivery method (gavage, injection or inhaled). With this design, we can estimate the effect of the drug and delivery method on blood pressure and then determine if the effect of drug depends on the delivery method.

A factorial design allows us to include more than one independent variable of interest. A very topical example would be sex. Within preclinical research, there is currently an embedded sex bias as researchers predominately study only one sex; typically males.^31 32^ This bias is apparent even when the disease of interest is a female prevalent disorder.^33^ Yet we know that for many diseases women and men differ in the prevalence, progression and severity of a disease and during treatment can experience significant differences in the side effects and efficacy.^34^ Recent research in the field of preclinical pain has found that where a sex difference existed, the treatment effect was predominately seen in the males and the authors argue that this indicates that the underlying mechanistic understanding is biased on the historic biased data and is skewed towards the biology of males.^35^ Several funding agencies have been encouraging researchers to study both sexes,^36^ and the US National Institute of Health (NIH) have mandated change and request that all studies funded by them include both sexes bar a few circumstances.^37^

Often researchers will argue that they will test the second sex later and that inclusion of both sexes will double the sample size of the study.^34^ The first argument is flawed; when you test the second sex later, you have no idea whether any differences seen in the estimated treatment effect between the two sexes are biologically meaningful or due to experimental variation unless you run both sexes simultaneously. The second argument is a misconception and relates to a lack of awareness of how factorial designs work. In the absence of an interaction, factorial designs are more sensitive, which is why McCarthy in 2015 suggested that you mirror your original design but change half the animals in your study to female.^38^ This sensitivity arises as the power for a main effect of treatment depends on the overall sample size per level of each factor as the effect of treatment is estimated across both sexes simultaneously.^39^ If the effect is very dependent on sex, the power will of course be impacted; however, at this point a significant biological insight has been obtained that was worth the loss of power. Resistance to the study of females has also arisen from the flawed belief that female animals are more variable due to the oestrous cycle, which has been disproven by a meta-analysis.^40^ Fundamentally, the focus on one sex has arisen from an interpretation of the Reduction element of the 3Rs framework to minimise the number of animals as far as possible within one single experiment. Using one sex and assuming the results will extrapolate is a consequence of this. This of course ignores the fact that in the breeding of the animals both sexes are produced at an equal rate. Furthermore, sex bias contributes both to the translation and reproducibility crises.^2 34^ They contribute to the latter when researchers fail to report the sex used, or if they allow sex to be an uncontrolled variable during the experiment, or if they fail to account for sex during the analysis when sex is a significant source of variation. The reproducibility crisis has led to a refinement in the definition of the Reduction element by the NC3Rs to “appropriately designed and analysed animal experiments that are robust and reproducible, and truly add to the knowledge base”.^41^ This indicates we need to review our interpretation of Reduction and focus beyond the N within a single experiment. Failure to include females is a missed opportunity to understand the biology on a crucial variable.

**Figure 3:**
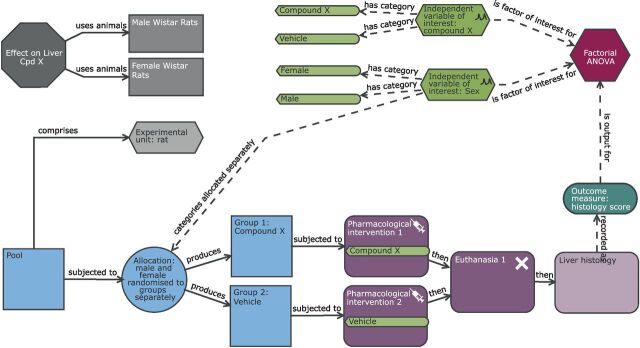
Schematic of a factorial design to study the effect of compound X on the histological score with sex as a second independent variable of interest. Compared with the completely randomised design (figure 2), the analysis now has two independent variables of interest (sex and treatment). The allocation also differs in that the assignment to treatment group happens for each level of sex independently. In this design, the experimental unit and observation unit is an individual Wistar rat and the biological unit is the Wistar rat which encompasses both male and female animals and represents the increased external validity of the study by the inclusion of both sexes.

Using a factorial design for the liver case study, with treatment and sex as factors (figure 3), we can assess in a single experiment the effect of the drug, the effect of sex and whether the effect of the drug depends on the sex. In line with the NIH guidelines, we include both sexes in the study but do not power the study to investigate where the treatment effect varies between sexes (ie, detect a sex by treatment interaction effect). This approach is appropriate as our primary focus is on whether there is a treatment effect and if there is a large difference in the treatment effect across the two sexes, we will be in the position to see this. Consequently, we use the same number of animals^38^ compared with the CRD, but the inference space has significantly improved and now represents the target population of interest for the compound. We also have integrated into the analysis a statistical test to assess whether the effect depends on sex. The EU is the individual animal which can be randomly assigned to any of the levels (male, female, vehicle or compound X) for the two factors of interest. The random assignment for the variable sex is driven by Mendelian inheritance while for the treatment assignment this would be implemented by the researcher. The OU is also the individual animal. The BU differs from the CRD (figure 2) as now it is Wistar rats which encompasses both sexes and consequently this experiment provides a larger inference space than the CRD.

### Randomised block design

A randomised block design (RBD) can be used to manage nuisance source of variability by including nuisance variables in the experimental design and analysis to account for them. These sources of variation may affect the measured results but are not of direct interest to the researcher. Examples of a nuisance variable could be litter, operator, instrument, batch, laboratory, time of day the experiment was run and so on. You can consider a RBD as a series of completely randomised experiments (mini-experiments) which form the blocks of the whole experiment.

**Figure 4:**
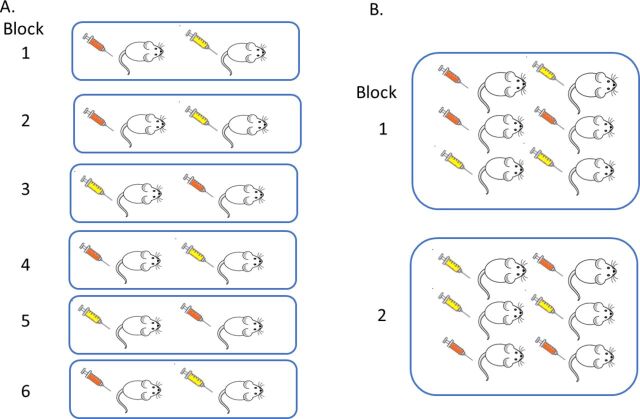
Understanding a randomised block design (RBD). In a RBD, each block can be considered a mini-experiment. Within a block (shown in this schematic as a group of mice within a blue square), the experimental unit (EU, shown as a mouse) is randomly assigned to a treatment (shown as a syringe which is coloured orange or yellow dependent on the treatment assigned) and all treatment levels are represented. (A) represents an experiment with six blocks with only one experimental unit per treatment level per block. (B) represents an experiment with two blocks with replication within a block resulting in multiple EUs per treatment level per block (B).

In a block design, within a block there is standardisation but between blocks there is variation. To account for this structure, the EUs are randomly assigned to treatment conditions within each block separately. This process has the impact that the variability within a block is less than the variability between blocks. Consequently, in the analysis, power is increased as the effect of interest is assessed ‘within block’ against the within-block variability rather than the variability in the whole experiment. This strategy will only be effective if the variability within the block is actually lower than the variability between blocks when the experiment is performed. There are two options for a RBD, to have either a single EU or multiple EUs for each treatment per block (figure 4). In terms of estimating the average effect across blocks, there is greater impact on power by increasing the number of blocks than increasing the replication within a block. The design selected will have a practical element. If the block is litter or cage, replication will naturally arise.

It has been argued that our reliance on CRD along with highly standardised environments is contributing to the reproducibility crisis.^6^ When reproducibility issues were first raised with in vivo studies, there was a call for greater standardisation.^42–44^ It was believed that standardisation of procedures, environmental conditions, genetic background and testing conditions would reduce variability, thereby enhance power and with well-defined testing conditions, environment and procedure support reproducibility. The drivers behind this approach were twofold. First was to remove potential confounders from the data to ensure that the only difference between the control and treated groups was the treatment. The second driver was a focus on reducing the variation in the data resulting in fewer animals being needed to detect a defined effect size of interest. If we relate the resulting design to a clinical study, this would be equivalent to testing a treatment on 30-year-old identical twin brothers who all live in the same village, followed the same career path and now live a life with the same monotonous diet and exercise routine.^45^ Reflecting on this raises several questions: Why would this observed effect in such a standardised situation be representative of a population? Why do we expect an effect observed in this standardised situation, with artificially low variation, to translate to a meaningful effect once normal biological variation is present?

The problem with the standardisation strategy is that living organisms are highly responsive to the environment with phenotypic changes with both long-term (eg, development) and short-term (eg, acclimation) duration.^46^ This ability, called phenotypic plasticity, is an evolutionary adaption for survival and ensures optimal fit to the current environment. Without phenotypic plasticity, there would be no treatment effect. Numerous experiments^47–50^ have highlighted the responsiveness of animals to the environment and that the treatment effect can be dependent on the environment within which the experiment was conducted. For some treatments, the effect could be robust and only depend on the nature, duration and intensity of the treatment. However, it is more likely that the treatment effect will be context dependent and will also depend on the animal’s current phenotype which arises from its history, genotype and the experimental context.^6^ These observations have led to the realisation that in vivo studies in highly standardised environments may identify idiosyncratic effects with low reproducibility. The further push to standardise to attempt ‘to increase reproducibility at the expense of external validity’ has been called the standardisation fallacy.^51–56^

Instead of a focus on standardisation to minimise variation, it has been argued that we should embrace variability to ensure conclusions from studies are more representative and thereby improve the generalisability and hence the reproducibility.^6^ While the initial focus on the use of RBD was to account for nuisance variables that could not be avoided to increase power, the growing awareness of the reproducibility crisis and the issues with standardisation had led to RBD being considered one of the key tools to improve the external validity.^6^ With a RBD, the generalisability of the estimated treatment effect is increased as the effect is estimated from blocks where systematic variation is introduced. The blocking factor is a mechanism to introduce variation while having standardisation within a block.

**Figure 5:**
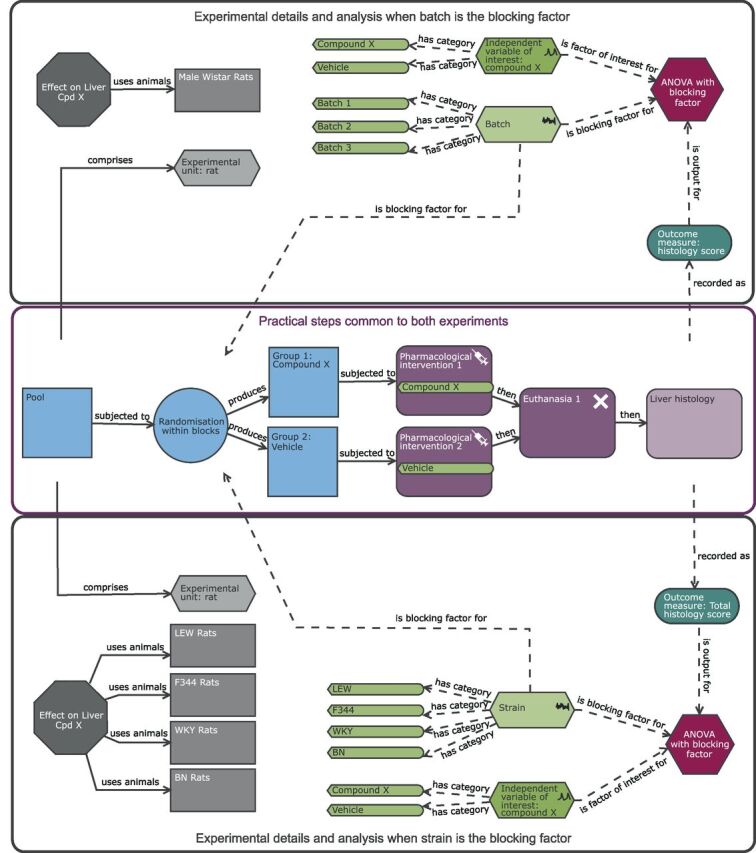
Schematic of a randomised block design to study the effect of compound X on the histological score with either batch (upper black pane) or strain (lower black pane) as a blocking factor. The purple section highlights the common practical steps. In the upper black pane, the experiment is split into a number of batches and the analysis includes a blocking factor ‘batch’. The blocking factor has three levels (batch 1, batch 2 and batch 3). The diagram highlights that the experimental rats are now randomly allocated to the treatment group within each batch. In this design, the experimental unit (EU), biological unit (BU) and observation unit (OU) is the Wistar rat. The estimated effect is more reproducible than the completely randomised design (figure 2) as it is the average treatment effect across the three batches. In the lower black pane, the experiment includes multiple strains and the analysis now includes a blocking factor ‘strain’. The blocking factor has four levels (LEW, F344, WKY and BN) allowing the estimation of the treatment effect across four outbred strains simultaneously. As this is a block design, rather than a factorial arrangement, the analysis does not explore how the treatment effect depends on strain but assesses the treatment effect relative to the pooled variability estimate that is a combination of the within-strain and treatment by strain variability. In this design, the EU and OU is the individual rat of a certain strain while the BU is now rat and generalisability has been increased as the inference space includes multiple strains.

In the liver case study, with a RBD we can explore the effect of the drug across a blocking factor to increase the generalisability of the results. One option would be to introduce variation by including batch as a blocking factor (figure 5) rather than running the complete experiment in one single batch in time as in the CRD (figure 2). A batch would be an independent run of the experiment and at a minimum would differ in time and batch of rats but also could include separate reagents, operator and so on. With batch as a blocking factor, we would have confidence that we would be able to reproduce the results for that environment as standardised by the metadata for that facility (eg, diet, cage conditions etc) as the estimated treatment effect is averaged over the environmental variation that could occur within that standardised environment.^57^ The most extreme version of this design would have in each batch one animal per treatment level (figure 4A). The experiment would be more time consuming; it would, however, maximise power to detect the average treatment effect as the replication is focused on different batches which leads to high confidence that the estimated effect is reproducible for this laboratory’s conditions and processes. Figure 5 represents a pragmatic solution with some replication within batch (figure 4B) which enables batch variation to be considered but avoids a long, complicated experiment. In this design, the EU, OU and BU are all the male Wistar rats. The estimated effect will be more reproducible as the design results in the estimate being the average treatment effect across the three batches and the significance of the effect is benchmarked relative to the variability within each batch and variation of the treatment effect across batches.

The question is what n should be used within a block and how many blocks are needed. This topic was explored for four syngeneic tumour models^57^ using simulations and growth characteristics seen within a laboratory. The authors recommended a minimum of three batches to ensure the average estimate encompassed three independent environments. The optimum number of animals per batch depended on the variability of the individual model. The simulations highlighted that there was an increase in the number of animals used in the multi-batch design; however, there was reduction in the number of animals used compared with the number used if you had repeated a standard CRD experiment.^57^

For the rat case study with batches as the blocking factor, we can determine the n to use using the Kastenbaum *et al*^58^ series of tables which report the maximum standardised effect size that will be detected for various block designs. Using these tables, which gives the best case scenario, we would be able to detect a standardised effect size of 1.44 with 3 batches and 3 EU per batch per treatment level (k treatments=2, b blocks=3, n observations per treatment group within a block=3, significance threshold=0.05 and target power=0.8). This design in total uses 18 animals which is slightly larger than the CRD (n=16). However, it is an estimate across three independent batches and the researcher will have greater confidence in the reproducibility of the observed effect.

An alternative approach to this experiment would be including strain as a blocking factor (figure 5). The inclusion of four different strains allows us to estimate the average treatment effect across the four strains and the estimated effect will be benchmarked against the variability within the strains and the variability of the treatment effect across the strains tested. In this design, the four strains chosen should be chosen as a random sample from a population of strains. As there is replication within each strain, a secondary analysis could assess how consistent the treatment effect is and whether it depends on strain.^6^ This analysis is equivalent to that conducted with a factorial design where the primary goal is to know the effect for each strain, if studying the effect of strain is the goal the initial hypothesis should state this and the experiment be powered for that objective. Strain provides an example of a variable that could be a factor of interest or a nuisance variable depending on the research goals.

In this block design, the EU and OU is the individual rat of a certain strain while the BU is now rat and generalisability has been increased as the inference space includes multiple strains. Using the Kastenbaum *et al*^58^ series of tables, we would be able to detect a maximum standardised effect size of 1.22 with 4 strains and 3 EU per strain per treatment using a N of 24 rats (k treatments=2, b blocks=4, n observations per treatment group within a block=3, significance threshold=0.05 and target power=0.8). Compared with the CRD, there is a 30% increase in the number of animals needed, but the inference space is significantly enhanced and we would have data to understand the treatment effect across four strains rather than one alone.

We could reduce the number of rats within a block to two (total n=16) and detect the standardised effect size of 1.6 (k treatments=2, b blocks=4, N observations per treatment group within a block=2, significance threshold=0.05 and target power=0.8), but have insufficient replicates to assess how the treatment effect interacted with strain. This approach uses the same total number of animals as the CRD but would have a significantly enhanced inference space.

### Hierarchical nested designs

Replication within an experiment is often misunderstood, especially when the design is hierarchically nested, leading to a poor focus of resources and inappropriate statistical analysis.^14^ These errors will contribute to the reproducibility crisis. Replication, in hierarchical nested designs, can be divided into two types: absolute replication (which increases the sample size n) and replication which can lead to pseudoreplication (a process artificially inflating the number of samples when the statistical analysis used was inappropriate for the design).^14^ In a survey of in vivo studies where experimental interventions were applied at one level (eg, parent) but the effect examined at another level (eg, offspring), they found that only 22% of studies had genuine replication, 50% had pseudoreplication and 32% provided insufficient information to assess what was done.^14^

Hierarchical nested designs are common in biomedical research. It can be considered a form of subsampling where an EU is sampled multiple times, typically to get a more accurate measure of the EU’s response. For example, blood pressure readings are very sensitive to the environment and consequently studies on rodents will typically take multiple readings per day for each animal.^59^ In this situation, the within-EU readings are not independent; readings collected on the same day for a rodent will be more similar than the readings between rodents. If these data are just pooled for a treatment group and analysed with a classic Student t-test which assumes independent readings, then the correlation will lead to underestimation of the population variability which will result in an inflated estimate of statistical significance and thus will lead to type I errors.^60^ Considering the EU, OU and BU for your designs is a powerful way to help identify when hierarchical structure exists and then statistical solutions can be identified.

**Figure 6:**
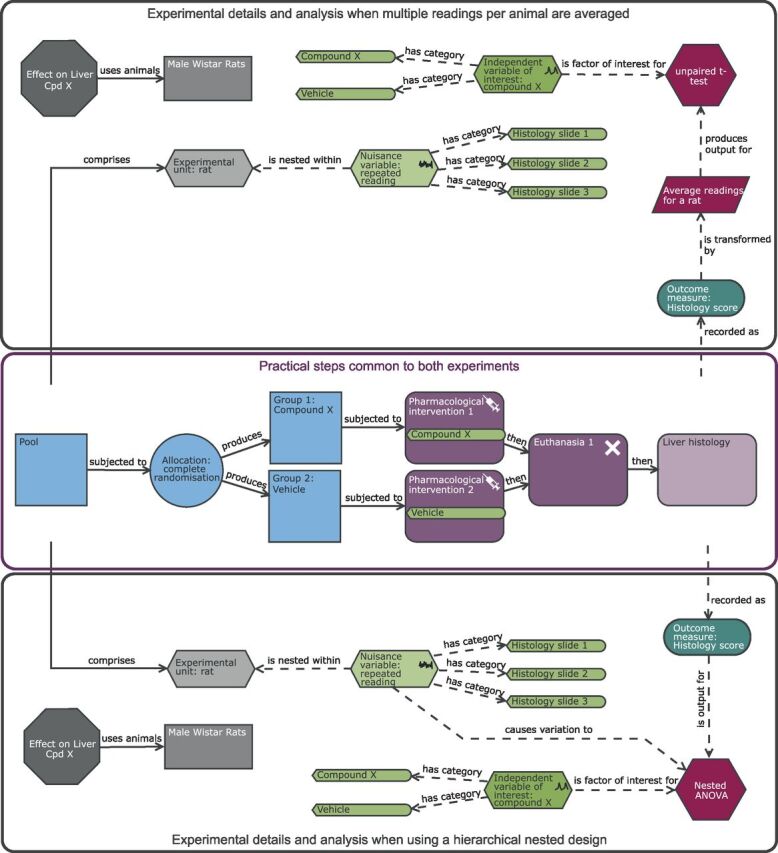
Schematic of a completely randomised design (CRD) to study the effect of compound X on the histological score with multiple readings per rat. Compared with the original CRD (figure 2), there is the additional nuisance variable ‘repeat reading’ which is nested within the experimental unit (EU). To avoid pseudoreplication, multiple readings are managed either through an averaging strategy (upper black pane) or through accounting for the structure in the analysis (lower black pane). The purple section highlights the common practical steps. With this design, the EU is the rat, the observation unit is the histological slide for a rat, while the biological unit is the male Wistar rat and is exploring the effect of treatment on the average reading for a rat. When the hierarchical nested structure is managed through the statistical analysis, then the nuisance variable is linked directly to the analysis node. Avoiding the summary metric can increase power, and the benefit will depend on how many readings are collected per animal and how variable the data are across slides.

An approach frequently used to manage sub-sampling is a summary statistics approach (ie, you average the readings). For example, for the blood pressure measure, you would average the readings collected for a rat and this would allow you use to use this summary metric as the reading to represent the EU in the analysis. This approach can be applied to an alternative version of the rat liver study where instead of working with the single histological score for each rat we could treat each reading on a histological slide as an OU replication for the rat (figure 6). We can calculate an average as a summary metric for each rat. This slightly changes the hypothesis being tested by the statistical test. For a Student’s t-test comparing a vehicle and a treatment group, with individual readings you are asking is there a difference in the group means of the individual readings. If instead you are working with averages you are assessing for a difference in the group mean of the average readings. The use of the average would be a way to improve the power compared with a single histology reading per rat as it reduces the impact of the within rat variability. In this design, using the standardised effect concept, we would need eight animals per treatment group to detect a SD difference of 1.5 units in the average reading for a rat with a power of 0.8. This initially seems equivalent to the power returned for the CRD (figure 2); however, if there is variation across the slides, the variability between the readings would be higher for a study using only a single histological score per rat than for an experiment that used an average of the OUs for a rat. Consequently, for the same 1.5-unit change as a standardised effect, the underlying treatment effect that the experiment could detect would be larger for the experiment with multiple readings per animal.

Using a summary metric is not optimal in terms of statistical power as it ignores intra-subject variance.^61^ Given the priority to minimise animal usage, it is recommended that the more sophisticated analytical techniques are embraced. Furthermore, an advantage of analysing the raw data is that you can explore the sources of variance and understand where resources should be allocated to maximise power in future experiments.^62^ For example, if you find that the within-animal variance is high and the cost to collect additional OU per EU is low, then there is significant value in increasing the number of OUs. The decision to analyse the raw data in the rat liver study can be seen visually in an EDA diagram (figure 6 lower pane) as the nuisance variance ‘repeated reading’ links directly to the analysis node. In these designs, the variance and number of readings at each level influence the statistical power and increasing the replication at a higher level has more influence than increasing it at a lower hierarchical level.^59^ Without knowledge of how variance varies across the hierarchy, we cannot predict the impact on power. In this case, we would design this case study based on the summary metric (using the figure 6 power justification) but knowing that the hierarchical analysis using all the data could be more sensitive. Once an initial experiment has been conducted, a variance analysis and subsequent exploration of power can be conducted.

### Serial/repeated measures design

Frequently, data are collected serially across all subjects either taking multiple measures of the same variable under different conditions or over two or more time periods. These within-subject designs are used in order to assess trends over time or to increase power as you are able to partition the within-animal and between-animal variability that treatment effects are assessed against. Examples include tumour growth curves, or monitoring heart rate and respiratory parameters after exposure to an intervention/treatment for a period of time, or the glucose tolerance test which tracks blood glucose concentration after exposure to glucose treatment. These repeated readings differ from replication discussed earlier in the hierarchical nested designs (figure 6); in a repeated-measures design, these repeat readings are indexed by a factor of interest (eg, time or independent treatment exposure) and are consistent across subjects.

**Figure 7:**
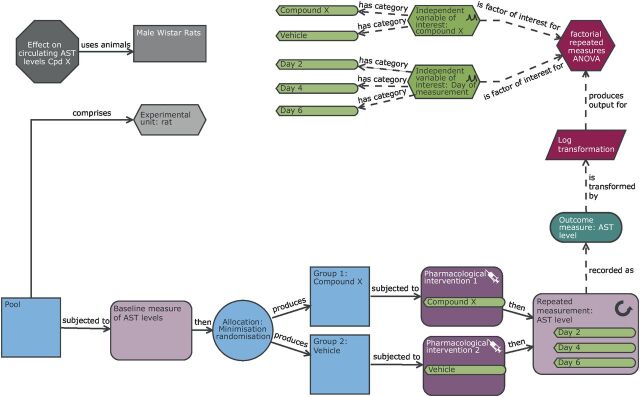
Schematic of a repeat measure design to study the effect of compound X on the circulating aspartate transaminase (AST) levels with time. In comparison with the original completely randomised design (figure 2), the outcome measure here is circulating AST levels which is monitored over multiple days. As this design is interested in how these levels change following exposure, there is a repeated measurement node in the design indicating that the readings are taken on days 2, 4 and 6. Furthermore, within the practical steps, we can see a baseline measure of AST level is taken prior to exposure and this is used in the allocation process to minimise potential differences in this variable at the group level. The AST variable has an underlying log normal transformation; consequently in the analysis section of the diagram, there is a transformation node to highlight the log10 transformation necessary to meet the statistical analysis assumptions of normality. In this design, the experimental unit, observation unit and biological unit is the rat and the experiment will be testing the hypotheses of whether the AST level depends on compound exposure, the day of measurement and whether the effect of compound varies with time (day 2 to day 6).

If we revisit the rat case example, the designs have directly assessed the impact of the compound X on the liver through a terminal histology assessment. If we were interested in exploring the temporal effect of the compound, this approach would need multiple groups for vehicle and treatment to sample at each time point. To enable serial sampling, we could use a proxy for liver damage and monitor the aspartate transaminase (AST) enzyme level in the circulating blood via microsampling (figure 7). In this design, we could collect a baseline measure for AST and allocate EUs to treatment by minimisation instead of randomisation.^63^ Minimisation is a method of randomisation that allocates EUs to treatment groups while ensuring that no systematic difference exists between the groups for one or more nuisance variables.^64^ Examples of nuisance variables that could be used include tumour volume at the start of dosing, baseline heart rate or baseline time spent in the centre in a behavioural assay. Freeware exists that can be used to implement such a strategy (eg, MinimPy^65^ or Minim^66^).

Data from serial measure experiments can be analysed with a repeated measures mixed models or through the calculation of summary statistics, such as the area under the curve, the slope from a regression analysis or the time to peak. With a summary metric, the analysis reverts to a simpler analysis pipeline as seen with the CRD. For this case study, if we summarise the data with the area under the curve to represent the toxicity load across the time course, then the power analysis reverts to that seen with the CRD: with a n of 8 per group, we would have 0.8 power to see a change equivalent to 1.5 SD in the summary metric.

The alternative repeated measures mixed model analysis strategy models the correlation and accounts for the lack of randomisation within an animal and thus should return an efficiency advantage and give a more nuanced analysis as the effects are explored with time. The power calculation is, however, more complex as mixed model analysis accounts for the correlated structure in the data. GLIMMPSE is a web-based freeware that has been developed with a mode (Guided Study Design) for researchers to calculate the statistical power of repeated measures studies.^67^ Using this tool, nine rats per group would be needed to return a power >0.8 to detect a difference between the means at any one time point equivalent to 1.5 SDs (method: Hotelling Lawley Trace, significance threshold 0.05, power=0.848, assuming no correlation between days as we have no data to assess this). With a pilot or related data, correlation could be assessed and the power analysis adjusted, as including a known correlation allows us to account for some of the variability in the data which should reduce the N required.

## Case study 2: milk production in dairy cows

To highlight different potential design features, we need to consider an alternative case study. The key biological question, in this case study, is to explore the effect of diet on milk production in dairy cows within a single farm. The arrangements at the farm allow the cows to feed individually. 68

### Completely randomised design

**Figure 8:**
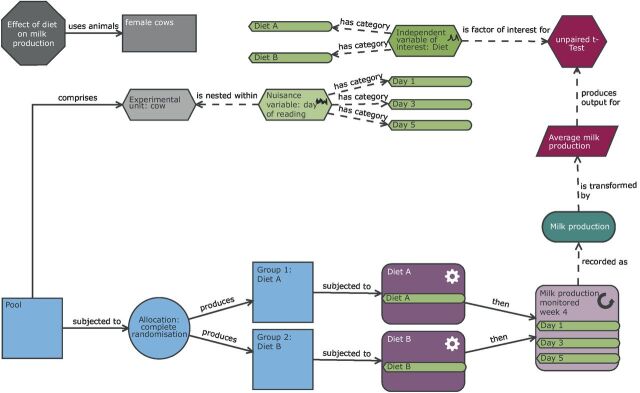
Schematic of a completely randomised design to study milk production in dairy cows. In this completely randomised design, there is one factor of interest (diet with two levels: diet A or diet B). The individual cows are randomly allocated to a diet and milk production subsequently measured three times in the last week of the following month (allowing several weeks for the animal to adjust to the new diet). In this design, the cow is the biological unit and the experimental unit. The observation unit is the measurement of milk production on a day for a cow. As shown in the diagram, to account for this repeat measure structure, the average milk production is calculated prior to the statistical analysis.

The advantages and disadvantages of CRD have been discussed previously. These would apply to using a CRD for the milk production study, and as in the rat study the EU would be the animal. In practice, this study would involve serially repeated measurements as the cows would be milked daily, and the average daily production over a suitable time period after initial acclimatisation to the diet would be used in the analysis (figure 8). Performing the study this way would likely involve a considerable number of animals. For a median sized effect of interest (Cohen’s *d*=0.5),^24^ then we would need 65 cows per group and thus 130 cows in total (Russ Lenth Power Tool,^23^ SD 1, significance threshold=0.05, tails=two, effect size=0.5, power=0.80).

### Cross-over design

In a cross-over design, each participant will receive multiple treatments with a wash-out period between exposures and outcome measurement. The order in which the animals receive the treatment is randomised to account for potential temporal effects. This type of design relies on the measurement being non-terminal and the treatment effect being reversible. A wash-out period is a critical step to allow the participants to return back to baseline readings before exposure to the next treatment. The disadvantages of this design are a risk of carryover effects confounding the estimated treatment effect and the welfare implications of an individual animal experiencing multiple procedures. Lengthy wash-out periods are recommended to ensure that carryover effects, where the exposure to the first treatment impacts the response to the second treatment, are minimised. The advantage of a cross-over design is that each participant forms its own control group and this dramatically increases the power.

**Figure 9:**
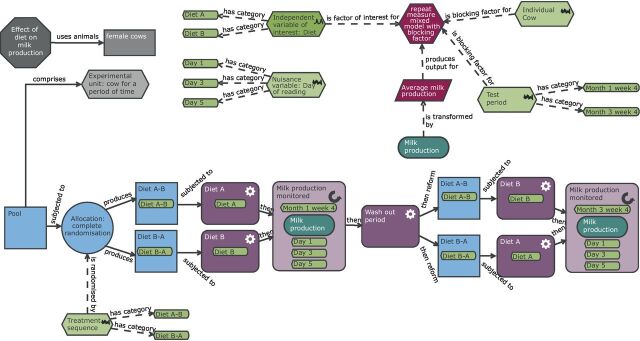
Schematic of a cross-over design to study milk production in dairy cows. Compared with the completely randomised design (CRD) (figure 8), the practical steps and the analysis have many more elements. In this design, each animal is exposed to both treatments with a wash-out period between them. The order of treatment is randomised to allow for any potential time effects to be accounted for in the analysis. The experimental unit is now the cow for a period of time and therefore the randomisation is either to treatment sequence A–B or B–A. The biological unit is the cow while the observation unit is the measurement of milk production on a day for a cow. To account for this repeat measure structure, the average milk production is calculated prior to the statistical analysis. The experiment is more complex than a CRD; however, far fewer animals are needed because each animal acts as its own control.

The cow milk production study could use a cross-over design (figure 9) as a diet intervention is considered reversible and a wash-out period equivalent to the diet exposure should be sufficient.^69^ The experiment would take longer than a CRD approach, but far fewer animals would be needed. Wellek and Blettner^70^ provide an efficiency conversion factor between a CRD and a two-period cross-over design with a factor of interest with two levels using the measurement error and the between-subject variance. With milk production in cows, we anticipate the variability in milk production between cows (between-subject variance) to be larger than the variability in cows’ daily milk production (within subject variance). If we make the assumption that the between-subject variance is twice as large as the within-subject variance, then using the conversion factor, we would need six times more cows in a CRD than a cross-over design. On that basis, the cross-over design would only need 11 cows per randomisation sequence, yielding 22-cow time periods in which milk production on a particular diet is assessed, compared with the 130 cows calculated previously for a two-diet comparison with a CRD. This reduction in animal usage is pronounced giving an ethical benefit and also a likely practical benefit in the experiments being easier to run.

## Discussion

To date, most scientists have implemented (knowingly or unknowingly) a CRD. The drivers towards this design are multifactorial and include lack of, or inadequate, experimental design training, minimal exposure to alternative designs, cultural norms, the historic 3R interpretation, misconceptions over N needed and a statistical skill gap.^71^ From our perspective, the largest driver towards the CRD is an interpretation of the 3R Reduction element, driving some scientists to use highly standardised experiments despite the risk of low generalisability. The limitations of the historic interpretation of the 3Rs to deliver robust experiments and failure to prioritise the scientific validity has led to calls for change in the framework surrounding experiments.^72 73^ These papers explore these issues and how we as a community can embrace these changes. The need to focus on the design and ensuring experiments are robust has led to the changes in the NC3Rs’ definition of Reduction.^41^ As a first step, ethical review boards/institute animal care and use committees need to focus on validity not just welfare.^72 73^

## Conclusion

The scientific process relies on a simplification of a complex biological process to generate a testing space where we can isolate cause and effect with the goal of incrementally unravelling the biological story. The focus on standardisation and the CRD leads to experiments which assess for a treatment effect within a narrow testing space and thus assess causality with limited generalisability. Furthermore, the publication process and focus on manuscripts as scientific measures of success encourage scientists to overstate their findings and generalise the results to a far wider population than that tested.^74 75^ This is a similar issue to the heavily criticised binary thinking seen with hypothesis testing and the p value where scientists conclude an effect is significant or not significant rather than considering the strength of evidence and the size of the effect.^76 77^ The reality is we do not prove. We collect evidence towards a biological understanding. There are many pitfalls in the experimental design process that are contributing to the reproducibility crisis. Even when these are avoided, there is no perfect experiment, they all have different strengths and weaknesses (eg, the narrow testing space in a standardised CRD study). As a community, we need to spend more time planning to ensure the design and analysis will answer our biological questions. It is also important when reporting to acknowledge the inherent limitations of the designs used. Then we will meet our ethical obligation to ensure our experiments are robust and are truly going to add to the knowledge base.
